# An Opt-Out Emergency Department Screening Intervention Leads to Major Increases in Diagnosis of Syphilis

**DOI:** 10.1093/ofid/ofae490

**Published:** 2024-09-10

**Authors:** Kimberly A Stanford, Joseph Mason, Eleanor Friedman, Aniruddha Hazra, Erin Augustine, John Schneider

**Affiliations:** Section of Emergency Medicine, University of Chicago, Chicago, Illinois, USA; Section of Infectious Diseases and Global Health, University of Chicago, Chicago, Illinois, USA; Section of Infectious Diseases and Global Health, University of Chicago, Chicago, Illinois, USA; Section of Infectious Diseases and Global Health, University of Chicago, Chicago, Illinois, USA; Section of Infectious Diseases and Global Health, University of Chicago, Chicago, Illinois, USA; Section of Infectious Diseases and Global Health, University of Chicago, Chicago, Illinois, USA

**Keywords:** congenital syphilis, emergency department, pregnancy, screening, syphilis

## Abstract

**Background:**

With rising rates of syphilis in the United States, novel strategies are needed to improve early diagnosis, particularly among priority populations such as pregnant people. As the primary source of healthcare for many communities with limited access to care, the emergency department (ED) visit represents a crucial opportunity for syphilis detection and congenital syphilis prevention.

**Methods:**

This pre–post design study examined all ED encounters for 2-year periods before and after implementation of an opt-out ED syphilis screening intervention in May 2019 at a large, urban, academic ED. Data on laboratory testing, syphilis status, and demographics were extracted from the medical record. Descriptive statistics and logistic regression were used to examine trends in syphilis screening and diagnosis.

**Results:**

Syphilis screening increased from 5209 (3.6%) to 37 289 (24.4%) encounters. Presumed active syphilis infection (PAI) increased 288%, from 161 patients (3.1% of those screened) to 624 (1.7%). The proportion of female PAI increased from 25.6% to 42.5%, despite no change in proportion of females screened. Post-intervention, 23.6% of PAI were tested for a urogenital sexually transmitted infection (STI) in the ED and 9.0% presented with symptoms of an STI by diagnosis code. Among pregnant people, screening increased from 5.9% to 49.9% of encounters, and syphilis diagnosis increased 750%, from 2 cases to 15.

**Conclusions:**

Opt-out ED syphilis screening led to a dramatic increase in screening and diagnosis, especially among pregnant individuals, a priority population for congenital syphilis prevention. Most individuals with syphilis did not have STI symptoms. Opt-out screening will be an important strategy in the effort to address the syphilis epidemic.

## Teaser

Opt-out emergency department syphilis screening led to major increases in both screening and diagnosis of syphilis among priority populations such as pregnant people and groups disproportionately affected by syphilis, most of whom presented without symptoms of a sexually transmitted infection.

Novel means of reaching individuals for syphilis screening and early diagnosis are urgently needed, considering the current epidemic of syphilis in the United States (US) [1], including a surge in congenital syphilis. Syphilis disproportionately affects communities of color and those with limited access to routine healthcare [1]. In 2022, almost 88% of congenital syphilis cases were born to persons who were not tested or received late diagnoses, and >37% to persons without prenatal care [2]. The emergency department (ED) is the default source of care for many individuals in underserved communities [3], and thus the ED visit is a critical opportunity for routine opt-out screening programs [4]. For this reason, ED human immunodeficiency virus (HIV) screening has been recommended for almost 2 decades [5], with a preponderance of evidence now available to support the success of these programs in diagnosing HIV [6] and the cost-effectiveness of ED HIV screening [7]. The National Academies of Sciences, Engineering, and Medicine [8] and the Centers for Disease Control and Prevention (CDC) [2] have more recently called for syphilis screening in nontraditional locations, including the ED. A growing body of literature supports targeted ED syphilis screening based on symptoms or other clinical factors such as pregnancy [9–13]; however, these models may fail to identify asymptomatic individuals with syphilis who do not meet these targeted screening criteria, such as women of reproductive age, thus limiting their potential to affect the syphilis epidemic. Nontargeted, opt-out screening programs [14], also known as universal screening programs, have increasingly been recommended in EDs [15], but to date there is limited evidence as to their effectiveness. A pilot study in 2020 [14] presented basic demographic trends from the first 7 months of a novel universal ED syphilis screening program, implemented in a large, urban hospital. The authors now undertake a much more in-depth analysis at the same site, comparing the screening and diagnosis rates found during the first 2 years of syphilis screening to the baseline rates in the prior 2 years, with more detailed examination of demographic and clinical trends, allowing for an evaluation of the impact of the screening program on screening and diagnosis of syphilis in the ED. Understanding the effect of interventions that screen large numbers of individuals in communities with high syphilis prevalence represents a crucial step for future efforts to control syphilis [8].

## METHODS

### Study Setting and Population

This study took place in a large, urban, adult ED in Chicago with a Level 1 trauma center. In May 2019, the ED implemented a routine, opt-out syphilis screening program, utilizing a reverse sequence algorithm for syphilis testing, as has been described in previous work [14]. For ease of implementation, the syphilis screening model utilized existing electronic health record (EHR) infrastructure for HIV screening based on CDC guidelines [5]: patients are flagged for screening if they are between ages 18 and 64 years, have no documented diagnosis of HIV in the EHR, and have not been screened for HIV within the past 12 months. In addition, tests can be ordered on any patient based on clinical decision making, even if the EHR does not suggest screening. All positive test results were reported to the health department, and a team from the Section of Infectious Diseases contacted all patients with positive tests to determine clinical history and referred them when appropriate for treatment within the institution or at affiliated clinics if needed.

**Figure 1.**
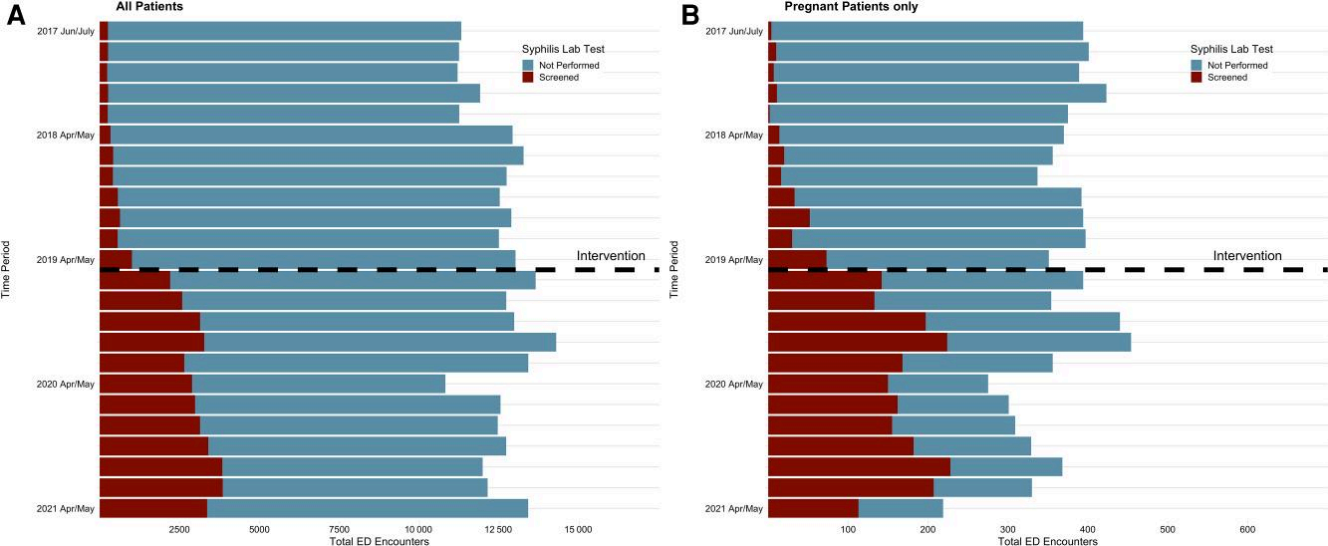
Proportion of emergency department (ED) encounters screened for syphilis pre- and post-intervention among all patients (*A*) and pregnant patients (*B*).

Using a pre–post study design, data were collected for all patients over age 18 visiting the ED during the 2-year periods before (1 June 2017 to 30 May 2019) and after (1 June 2019 to 30 May 2021) implementation of the opt-out syphilis screening program (Figure 1). Because the reason for syphilis screening could not be ascertained (ie, if due to routine screening or clinical concern), repeat encounters with syphilis screening within a 12-month time period, those among patients with HIV, and those older than age 65 were retained.

### Outcome Measures

The primary outcome for this analysis was presumed active syphilis infection (PAI), defined as any patients with positive syphilis immunoglobulin G and reactive rapid plasma reagin (RPR) or *Treponema pallidum* particle agglutination (TP-PA) without documented history of past positive testing and/or treatment as determined by clinical history documented in the EHR or comparison with records of prior syphilis testing or treatment obtained from the health department, as well as any patient with RPR ≥1:8 for whom no clinical or health department history was available. The inclusion of TP-PA–positive patients is an expansion of prior case definitions [14], as clinical history for TP-PA–positive patients was not included in the dataset utilized in earlier work. Any patient with positive TP-PA for whom clinical history was not available was considered not PAI for the purposes of this study.

### Covariates

For each ED encounter, demographics (age, gender, race/ethnicity, insurance type) and clinical information were extracted, including acuity level as measured by the Emergency Severity Index, presence of any additional blood draws other than HIV or syphilis, testing for urogenital sexually transmitted infections (STIs), including gonorrhea, chlamydia, or trichomoniasis, pregnancy testing and results, *International Classification of Diseases, Tenth Revision* (*ICD-10*) diagnosis codes, and HIV and syphilis screening data (results of current testing and previous clinical history). Laboratory testing was matched to ED encounters using a 2-day buffer. *ICD-10* codes were used to classify the reason for each patient encounter. Codes that would have been added to the encounter after the results of laboratory testing, such as codes for asymptomatic stages of syphilis, were excluded.

### Statistical Analysis

Descriptive statistics were used to analyze the distribution of demographic and clinical characteristics of patients in the study sample before and after implementation of the screening program. Bivariate and multivariate logistic regression models were created to examine associations, reporting odds ratios (ORs) (or adjusted odds ratios [aORs]) and 95% confidence intervals (CIs). All data analysis was performed using R (version 4.0.3; R Core Team). Subgroup analyses of women of reproductive age (defined as under age 50) and pregnant women (defined by a positive serum or urine pregnancy test during the ED visit) were performed.

### Patient Consent Statement

This study was approved by the University of Chicago Institutional Review Board (IRB). This study received a waiver of patient consent from the IRB.

## RESULTS

A total of 299 651 ED encounters occurred during the 4-year study period. Pre-intervention, 5209 of 146 644 (3.6%) encounters included syphilis screening, which increased to 37 289 of 153 007 (24.4%) encounters post-intervention (Table 1). Screening increased from 3.1% to 29.4% among patients under age 65, the intended screening population, although the exact proportion of eligible patients screened (able to opt-out, not known to be living with HIV) is unable to be ascertained from the data. There was a 288% change in PAI from pre- to post-intervention, with an increase from 161 syphilis cases (3.1% of those screened) to 624 (1.7%). The demographic distribution of all ED encounters was similar in terms of age, sex, race/ethnicity, and insurance type across both time periods. The sex and insurance types of the population screened for syphilis were similar pre- and post-intervention, but there was an increase in the proportion of patients screened in the non-Hispanic White (4.6% pre, 6.4% post) and Hispanic (2.6% pre, 4.3% post) groups. While ages 25–34 had the highest proportion of screening in both periods (28.6% pre, 25.0% post), screening was more evenly distributed among age groups postintervention, with increases in screening proportion observed among patients aged 35–64 years. Overall, in the post-intervention period, the number needed to screen was approximately 75 individuals screened to detect one PAI.

**Table 1.** Demographic and Clinical Characteristics of All Emergency Department Encounters, Patients Screened for Syphilis, and Presumed Active Syphilis Infection Pre- and Post-intervention

Characteristic	Encounters by Time Period, No. (%)					
	Pre-intervention			Post-intervention		
	Total ED Encounters	Screened for Syphilis	PAI	Total ED Encounters	Screened for Syphilis	PAI
	(n = 146 644)	(n = 5209)	(n = 161)	(n = 153 007)	(n = 37 289)	(n = 624)
Demographic characteristics						
Sex						
Female	90 863 (62.0)	3165 (60.8)	41 (25.5)	88 764 (58.0)	22 041 (59.1)	265 (42.5)
Male	55 724 (38.0)	2044 (39.2)	120 (74.5)	64 213 (42.0)	15 248 (40.9)	359 (57.5)
Unknown	57 (0.0)	0 (0.0)	0.0 (0.0)	30 (0.0)	0 (0.0)	0 (0.0)
Race/Ethnicity						
Non-Hispanic Black	123 186 (84.0)	4718 (90.6)	152 (94.4)	129 220 (84.5)	32 094 (86.1)	589 (94.4)
Non-Hispanic White	12 675 (8.6)	240 (4.6)	2 (1.2)	11 396 (7.4)	2401 (6.4)	8 (1.3)
Hispanic	5118 (3.5)	133 (2.6)	2 (1.2)	5757 (3.8)	1618 (4.3)	15 (2.4)
Other/Unknown	5665 (3.9)	118 (2.3)	5 (3.1)	6634 (4.3)	1176 (3.2)	12 (1.9)
Age, y						
18–24	24 785 (16.9)	1597 (30.7)	35 (21.7)	23 515 (15.4)	6415 (17.2)	79 (12.7)
25–34	31 926 (21.8)	1492 (28.6)	51 (31.7)	33 343 (21.8)	9338 (25.0)	167 (26.8)
35–44	20 411 (13.9)	609 (11.7)	17 (10.6)	21 795 (14.2)	6413 (17.2)	82 (13.1)
45–54	19 975 (13.6)	408 (9.2)	25 (15.5)	20 306 (13.3)	6446 (17.0)	92 (14.7)
55–64	21 377 (14.6)	425 (8.2)	16 (9.9)	22 906 (15.0)	7374 (19.8)	156 (25.0)
≥65	28 170 (19.2)	606 (11.6)	17 (10.6)	31 142 (20.4)	1413 (3.8)	48 (7.7)
Insurance^a^						
Medicaid	62 561 (42.7)	2803 (53.8)	97 (60.2)	68 342 (44.7)	19 874 (53.3)	395 (63.3)
Medicare	36 876 (25.1)	808 (15.5)	23 (14.3)	40 750 (26.6)	5465 (14.7)	98 (15.7)
Private	30 349 (20.7)	805 (15.5)	18 (11.2)	28 716 (18.8)	8213 (22.0)	60 (9.6)
Other	16 858 (11.5)	793 (15.2)	23 (14.3)	15 199 (9.9)	3737 (10.0)	71 (11.4)
Clinical characteristics						
Tested for gonorrhea, chlamydia, or trichomoniasis						
Yes	7623 (5.2)	2801 (53.8)	77 (47.8)	7054 (4.6)	5129 (13.8)	147 (23.6)
No	139 021 (94.8)	2408 (46.2)	84 (52.2)	145 953 (95.4)	32 160 (86.2)	477 (76.4)
Presenting symptoms by *ICD-10* code						
STI related	3952 (2.9)	1329 (26.8)	28 (18.2)	3774 (2.7)	2258 (6.5)	51 (9.0)
Opioid use	2590 (1.9)	134 (2.7)	6 (3.9)	3788 (2.7)	1125 (3.2)	32 (5.6)
Any substance use	6956 (5.1)	373 (7.5)	20 (13.0)	9545 (6.8)	3298 (9.5)	97 (17.1)
Abdominal and pelvic pain	6334 (4.7)	548 (11.0)	8 (5.2)	6434 (4.6)	2855 (8.2)	26 (4.6)
Rashes	2059 (1.5)	216 (4.3)	19 (12.3)	1828 (1.3)	514 (1.5)	28 (4.9)
Flu-like illness	3208 (2.4)	79 (1.6)	2 (1.3)	2879 (2.1)	715 (2.1)	7 (1.2)
Altered mental status	5234 (3.9)	436 (8.8)	12 (7.8)	7598 (5.4)	1935 (5.6)	52 (9.2)
Pregnancy	5356 (4.0)	369 (7.4)	2 (1.3)	4782 (3.4)	2028 (5.9)	16 (2.8)
Additional blood draw^b^						
Yes	86 451 (59.0)	3225 (61.9)	109 (67.7)	93 150 (60.9)	30 312 (81.3)	516 (82.7)
No	60 193 (41.0)	1984 (38.1)	52 (32.3)	59 857 (39.1)	6977 (18.7)	108 (17.3)
Acuity level by Emergency Severity Index						
1	1482 (1.0)	102 (2.0)	0 (0.0)	2451 (1.6)	677 (1.8)	19 (3.0)
2	42 542 (29.0)	1254 (24.1)	51 (31.7)	44 769 (29.3)	11 981 (32.1)	185 (29.6)
3	54 301 (37.0)	1863 (35.8)	49 (30.4)	59 575 (38.9)	16 654 (44.7)	259 (41.5)
4	33 461 (22.8)	1552 (29.8)	50 (31.1)	28 983 (18.9)	5133 (13.8)	104 (16.7)
5	2945 (2.0)	73 (1.4)	7 (4.3)	2517 (1.6)	232 (0.6)	3 (0.5)
Unknown	11 913 (8.1)	365 (7.0)	4 (2.5)	14 712 (9.6)	2612 (7.0)	54 (8.7)
HIV testing						
Not tested for HIV	134 539 (91.8)	907 (17.4)	51 (31.7)	113 648 (74.3)	1431 (3.8)	61 (9.8)
HIV negative	11 810 (8.1)	4196 (80.6)	86 (53.4)	38 566 (25.2)	35 380 (94.9)	477 (76.4)
Known HIV infection	235 (0.2)	79 (1.5)	17 (10.6)	674 (0.4)	401 (1.1)	62 (9.9)
New diagnosis of HIV	50 (0.0)	27 (0.5)	7 (4.3)	83 (0.1)	77 (0.2)	24 (3.8)

Abbreviations: ED, emergency department; HIV, human immunodeficiency virus; *ICD-10*, *International Classification of Diseases, Tenth Revision*; PAI, presumed active syphilis infection; STI, sexually transmitted infection.

^a^For insurance, other includes self-pay and unknown insurance information.

^b^Additional blood draw refers to any laboratory test requiring a blood draw other than HIV or syphilis.

The proportion of encounters that included blood draws other than HIV or syphilis was similar in both periods (59.0% before, 60.9% after), but 81.3% of patients screened for syphilis in the postintervention period had additional blood draws, compared to 61.9% preintervention. This increase is reflected in the significant association between the presence of additional blood draws and the odds of syphilis screening, which increased postintervention (pre: aOR, 1.76 [95% CI, 1.63–1.91], *P* < .001; post: aOR, 5.79 [95% CI, 5.58–6.00], *P* < .001) (Table 2). The association between urogenital STI testing and odds of syphilis screening also decreased postintervention (pre: aOR, 33.29 [95% CI, 30.55–36.27], *P* < .001; post: aOR, 9.07 [95% CI, 8.84–9.71], *P* < .001), despite the number of urogenital STI tests remaining similar in both periods.

**Table 2.** Odds Ratios for Syphilis Screening by Demographic and Clinical Characteristics Pre- and Postintervention

Characteristic	Preintervention		Postintervention	
	Bivariate OR (95% CI)	Multivariate aOR (95% CI)	Bivariate OR (95% CI)	Multivariate aOR (95% CI)
Demographic characteristics				
Sex				
Female	1 (Reference)	NS^a^	1 (Reference)	1 (Reference)
Male	1.06 (.99–1.12)	NS	0.94*** (.92–.97)	1.00 (.98–1.03)
Race/Ethnicity				
Non-Hispanic Black	1 (Reference)	1 (Reference)	1 (Reference)	1 (Reference)
Non-Hispanic White	0.49*** (.43–.55)	0.80** (.69–.92)	0.81*** (.77–.85)	0.88*** (.83–.93)
Hispanic	0.67*** (.56–.80)	0.79* (.65–.95)	1.18*** (1.12–1.26)	1.04 (.98–1.11)
Other/Unknown	0.53*** (.44–.64)	0.87 (.71–1.06)	0.65*** (.61–.70)	0.81*** (.75–.87)
Age, y				
18–24	1 (Reference)	1 (Reference)	1 (Reference)	1 (Reference)
25–34	0.71*** (.66–.77)	0.87** (.80–.95)	1.04 (.99–1.08)	1.08*** (1.04–1.13)
35–44	0.44*** (.41–.49)	0.70*** (.63–.79)	1.11*** (1.07–1.16)	1.12*** (1.07–1.18)
45–54	0.36*** (.32–.40)	0.73*** (.64–.82)	1.21*** (1.16–1.26)	1.16*** (1.10–1.21)
55–64	0.30*** (.26–.33)	0.67*** (.59–.76)	1.27*** (1.22–1.32)	1.15*** (1.10–1.21)
≥65	0.32*** (.29–.35)	0.83* (.72–.96)	0.13*** (.12–.14)	0.11*** (.10–.12)
Insurance^b^				
Medicaid	1 (Reference)	1 (Reference)	1 (Reference)	1 (Reference)
Medicare	0.48*** (.44–.52)	0.85** (.76–.96)	0.38*** (.37–.39)	0.84*** (.80–.87)
Private	0.58*** (.54–.63)	0.86** (.79–.95)	0.98 (.95–1.01)	1.10*** (1.07–1.14)
Other	1.05 (.97–1.14)	1.00 (.91–1.10)	0.80*** (.76–.83)	1.08** (1.03–1.13)
Clinical characteristics				
Tested for gonorrhea, chlamydia, or trichomoniasis				
No	1 (Reference)	1 (Reference)	1 (Reference)	1 (Reference)
Yes	32.96*** (30.99–35.05)	33.29*** (30.55–36.27)	9.43*** (8.99–9.95)	9.07*** (8.84–9.71)
Presenting symptoms by *ICD-10* code^c^				
STI related	18.13*** (16.85–19.51)	1.97*** (1.78–2.17)	4.86*** (4.54–5.19)	1.85*** (1.70–2.02)
Opioid use	1.49*** (1.25–1.78)	1.67*** (1.37–2.17)	1.32*** (1.23–1.42)	1.06 (.98–1.14)
Any substance use	1.58*** (1.42–1.76)	1.67*** (1.48–1.90)	1.70*** (1.63–1.78)	1.17*** (1.12–1.23)
Abdominal and pelvic pain	2.76*** (2.51–3.02)	0.67*** (.60–.75)	2.60*** (2.47–2.73)	1.09** (1.02–1.16)
Rashes	3.28*** (2.84–3.78)	4.61*** (3.86–5.50)	1.22*** (1.10–1.35)	1.53*** (1.36–1.72)
Flu-like illness	0.68** (.54–.85)	0.81 (.63–1.04)	1.03 (.94–1.12)	NS
Altered mental status	2.60*** (2.35–2.88)	4.07*** (3.61–4.58)	1.06* (1.01–1.12)	0.98 (.92–1.04)
Pregnancy	2.09*** (1.87–2.33)	0.78*** (.69–.90)	2.36*** (2.23–2.51)	0.94 (.88–1.01)
Additional blood draw^d^				
No	1 (Reference)	1 (Reference)	1 (Reference)	1 (Reference)
Yes	1.12*** (1.06–1.18)	1.76*** (1.63–1.91)	3.61*** (3.51–3.71)	5.79*** (5.58–6.00)
Acuity level by Emergency Severity Index				
3	1 (Reference)	1 (Reference)	1 (Reference)	1 (Reference)
1	2.08*** (1.69–2.56)	2.24*** (1.94–3.06)	0.98 (.90–1.08)	0.84** (.76–.93)
2	0.86*** (.80–.92)	1.42*** (1.30–1.55)	0.94*** (.92–.97)	0.93*** (.90–.96)
4	1.37*** (1.28–1.47)	1.38*** (1.26–1.50)	0.56*** (.54–.57)	0.94* (.90–.99)
5	0.72** (.57–.91)	0.97 (.74–1.28)	0.26*** (.23–.30)	0.56*** (.48–.64)
Unknown	0.89* (.79–.99)	1.13 (.99–1.29)	0.56*** (.53–.58)	0.68*** (.65–.72)

Abbreviations: aOR, adjusted odds ratio; CI, confidence interval; *ICD-10*, *International Classification of Diseases, Tenth Revision*; NS, not significant; OR, odds ratio; STI, sexually transmitted infection.

^a^NS signifies that the variable was not significant at the bivariate level and was thus not included in multivariate model.

^b^For insurance, other includes self-pay and unknown insurance information.

^c^Reference group for each *ICD-10* category is encounters without *ICD-10* codes in that category.

^d^Additional blood draw refers to any laboratory test requiring a blood draw other than HIV or syphilis.

*P < .05

**P < .01

***P < .001.

There was an increase in the proportion of patients with PAI who were female (25.6% pre, 42.5% post) post-intervention, despite no change in the proportion of females screened. The proportion of Hispanic patients among PAI doubled (1.2% pre, 2.4% post), while the proportion of non-Hispanic Black and non-Hispanic White PAI remained the same. The largest changes in age distribution among PAI were an increase among ages 55 to 64 (9.9% pre, 25.0% post) and a decrease among ages 18 to 24 (21.7% pre, 12.7% post), which paralleled previously described changes in screening rates between intervention periods. There were marked differences in the demographic associations with PAI in the post-intervention period compared to pre-intervention. While males had higher odds of syphilis diagnosis in both periods (Table 3), the sex gap decreased post-intervention (pre: aOR, 3.50 [95% CI, 2.37–5.17], *P* < .001; post: aOR, 1.52 [95% CI, 1.31–1.84], *P* < .001). Older ages were also more highly associated with PAI in the post-intervention period, with individuals over age 65 having the highest odds of PAI (aOR, 3.24 [95% CI, 2.06–5.08], *P* < .001).

**Table 3.** Odds Ratios for Presumed Active Syphilis Infection by Demographic and Clinical Characteristics Pre- and Post-intervention

Characteristic	Pre-intervention		Post-intervention	
	Bivariate OR (95% CI)	Multivariate aOR (95% CI)	Bivariate OR (95% CI)	Multivariate aOR (95% CI)
Demographic characteristics				
Sex				
Female	1 (Reference)	1 (Reference)	1 (Reference)	1 (Reference)
Male	4.81*** (3.36–6.88)	3.50*** (2.37–5.17)	1.99*** (1.69–2.33)	1.55*** (1.31–1.84)
Race/Ethnicity				
Non-Hispanic Black	1 (Reference)	NS^a^	1 (Reference)	1 (Reference)
Non-Hispanic White	0.25 (.06–1.02)	NS	0.18*** (.09–.36)	0.24*** (.12–.49)
Hispanic	0.46 (.11–1.87)	NS	0.50** (.30–.83)	0.62 (.36–1.04)
Other/Unknown	1.32 (.53–3.28)	NS	0.55* (.31–.98)	0.59 (.33–1.07)
Age, y				
18–24	1 (Reference)	1 (Reference)	1 (Reference)	1 (Reference)
25–34	1.59* (1.03–2.46)	1.28 (.81–2.01)	1.47** (1.12–1.92)	1.43* (1.08–1.89)
35–44	1.30 (.72–2.34)	0.98 (.53–1.80)	1.04 (.76–1.42)	1.20 (.86–1.67)
45–54	2.52** (1.50–4.26)	1.48 (.84–2.62)	1.19 (.88–1.62)	1.51* (1.09–2.10)
55–64	1.77 (.97–3.24)	0.84 (.43–1.65)	1.76*** (1.34–2.31)	2.27*** (1.66–3.09)
≥65	1.33 (.74–2.40)	0.60 (.30–1.20)	2.86*** (1.99–4.11)	3.24*** (2.06–5.08)
Insurance^b^				
Medicaid	1 (Reference)	NS	1 (Reference)	1 (Reference)
Medicare	0.83 (.53–1.32)	NS	0.90 (.72–1.13)	0.70** (.54–.92)
Private	0.63 (.38–1.06)	NS	0.36*** (.28–.47)	0.49*** (.37–.65)
Other	0.84 (.53–1.33)	NS	0.96 (.74–1.23)	0.99 (.76–1.29)
Clinical characteristics				
Tested for gonorrhea, chlamydia, or trichomoniasis				
No	1 (Reference)	NS	1 (Reference)	1 (Reference)
Yes	0.77 (.57–1.06)	NS	1.95*** (1.62–2.35)	2.22*** (1.75–2.82)
Presenting symptoms by *ICD-10* code^c^				
STI related	0.60* (.40–.91)	0.88 (.56–1.40)	1.39* (1.04–1.85)	0.96 (.68–1.36)
Opioid use	1.50 (.65–3.46)	NS	1.78** (1.24–2.55)	1.11 (.75–1.64)
Any substance use	1.94** (1.20–3.13)	1.29 (.76–2.20)	1.94*** (1.56–2.42)	1.51** (1.18–1.91)
Abdominal and pelvic pain	0.43* (.21–.89)	1.00 (.46–2.15)	0.52** (.35–.77)	0.60* (.39–.91)
Rashes	3.31*** (2.01–5.46)	1.94* (1.11–3.40)	3.51*** (2.38–5.28)	2.64*** (1.72–4.04)
Flu-like illness	0.82 (.20–3.37)	NS	0.58 (.27–1.22)	NS
Altered mental status	0.90 (.49–1.63)	NS	1.69*** (1.27–2.26)	1.19 (.87–1.64)
Pregnancy	0.16* (.04–.64)	0.47 (.11–2.01)	0.45** (.27–.74)	0.65 (.39–1.11)
Additional blood draw^d^				
No	1 (Reference)	NS	1 (Reference)	NS
Yes	1.31 (.94–1.83)	NS	1.11 (.90–1.37)	NS
HIV testing				
Not tested for HIV	1 (Reference)	1 (Reference)	1 (Reference)	1 (Reference)
HIV negative	0.34*** (.24–.49)	0.34*** (.22–.50)	0.31*** (.23–.40)	0.37*** (.28–.50)
Known HIV infection	4.84*** (2.63–8.91)	3.06** (1.58–5.90)	4.21*** (2.89–6.11)	3.46*** (2.33–5.16)
New diagnosis of HIV	5.97*** (2.40–14.87)	2.66* (1.01–6.96)	10.24*** (5.92–17.70)	9.96*** (5.53–17.95)
Acuity level by Emergency Severity Index				
3	1 (Reference)	1 (Reference)	1 (Reference)	1 (Reference)
1	NS	NS	1.85* (1.15–2.97)	1.25 (.75–2.07)
2	1.59* (1.07–2.37)	1.08 (.69–1.69)	0.99 (.82–1.20)	0.80* (.66–.99)
4	1.23 (.82–1.83)	0.99 (.63–1.56)	1.31* (1.04–1.64)	1.05 (.81–1.34)
5	3.99** (1.74–9.16)	2.17 (.87–5.40)	0.85 (.27–2.66)	0.65 (.20–2.07)
Unknown	0.41 (.15–1.13)	0.44 (.15–1.26)	1.33 (.99–1.79)	1.18 (.87–1.60)

Abbreviations: aOR, adjusted odds ratio; CI, confidence interval; HIV, human immunodeficiency virus; *ICD-10*, *International Classification of Diseases, Tenth Revision*; OR, odds ratio; STI, sexually transmitted infection.

^a^NS signifies that the variable was not significant at the bivariate level and was thus not included in the multivariate model.

^b^For insurance, other includes self-pay and unknown insurance information.

^c^Reference group for each *ICD-10* category is encounters without *ICD-10* codes in that category.

^d^Additional blood draw refers to any laboratory test requiring a blood draw other than HIV or syphilis.

*P < .05

**P < .01

***P < .001.

Before opt-out screening, 47.8% of PAI were tested for a urogenital STI during the ED visit, and 18.2% were assigned STI-related diagnosis codes. Other common diagnosis categories pre-intervention included substance use (13.0%) and rash (12.3%). In contrast, post-intervention, 23.6% of PAI were tested for a urogenital STI, and 9.0% presented with STI symptoms, followed by substance use (17.1%), and altered mental status (9.2%). Post-intervention, the presence of urogenital STI testing was significantly associated with PAI in the adjusted analysis (aOR, 2.22 [95% CI, 1.75–2.82], *P* < .001); however, the diagnosis codes most strongly associated with PAI were rashes (aOR, 2.64 [95% CI, 1.72–4.04], *P* < .001). Post-intervention, 17.3% of PAI did not have any blood draw other than HIV and syphilis.

The proportion of PAI not screened for HIV (Table 1) decreased post-intervention (31.7% pre, 9.8% post), and the proportion of PAI among people with HIV (PWH) remained very similar (10.6% pre, 9.9% post), despite the screening algorithm theoretically excluding PWH from screening. There was a 343% increase in concurrent diagnoses of HIV and syphilis, from 7 (4.3% of PAI) pre-intervention, to 24 (3.8%) post-intervention. New diagnosis of HIV was highly associated with syphilis diagnosis post-intervention (aOR, 9.96 [95% CI, 5.53–17.95], *P* < .001), even more so than pre-intervention (aOR, 2.66 [95% CI, 1.01–6.96], *P* < .05).

While the screening program was designed to exclude patients over age 65 from routine screening in line with HIV screening guidelines, there was an increase in screening among this group post-intervention (606 pre, 1413 post). Similarly, PAI in patients over age 65 increased post-intervention (17 pre, 48 post). In both periods, the most common reason for ED presentation among patients over 65 screened for syphilis was altered mental status (40.1% pre, 28.0% post), followed by substance use (9.1% pre, 9.8% post). STI symptoms were uncommon in both periods (2.3% pre, 1.1% post). Post-intervention, no PAI over age 65 presented with STI symptoms, compared to 11.8% pre-intervention. Fewer PAI presented with altered mental status post-intervention (47.1% pre, 29.2% post).

### Pregnant Women and Women of Reproductive Age

There was an increase in screening among pregnant women (Table 4), with 272 of 4579 (5.9%) screened pre-intervention, compared to 2061 of 4129 (49.9%) post-intervention. Syphilis diagnoses increased by 750%, from 2 cases to 15, while the rate among the screened population of pregnant women remained unchanged (0.7% pre, 0.7% post). Distribution of race/ethnicity mirrored that of the larger population; however, pregnant women screened for syphilis predominantly utilized Medicaid (70.2% pre, 70.4% post), as did those testing positive (100% pre, 86.7% post). Post-intervention, 26.7% of pregnant PAI did not have another blood draw besides HIV and syphilis, and 66.7% were not tested for urogenital STIs. None of the pregnant PAI presented with STI symptoms (as compared to 50% pre-intervention), and 46.7% presented with abdominal or pelvic pain.

**Table 4.** Demographic and Clinical Characteristics of Emergency Department Encounters Among Pregnant Persons, Including All Encounters, Those Among Patients Screened for Syphilis, and Presumed Active Syphilis Infection Pre- and Post-intervention

Characteristic	Encounters by Time Period, No. (%)					
	Pre-intervention			Post-intervention		
	Total ED Encounters	Screened for Syphilis	PAI	Total ED Encounters	Screened for Syphilis	PAI
	(n = 4579)	(n = 272)	(n = 2)	(n = 4129)	(n = 2061)	(n = 15)
Demographic characteristics						
Race/Ethnicity						
Non-Hispanic Black	4249 (92.8)	255 (93.8)	2 (100.0)	3808 (92.2)	1896 (92.0)	15 (100.0)
Non-Hispanic White	96 (2.1)	4 (1.5)	0 (0.0)	78 (1.9)	29 (1.4)	0 (0.0)
Hispanic	152 (3.3)	9 (3.3)	0 (0.0)	144 (3.5)	84 (4.1)	0 (0.0)
Other/Unknown	82 (1.8)	4 (1.5)	0 (0.0)	99 (2.4)	52 (2.5)	0 (0.0)
Age, y						
18–24	1900 (41.5)	123 (45.2)	0 (0.0)	1638 (39.7)	802 (38.9)	6 (40.0)
25–34	2157 (47.1)	126 (46.3)	1 (50.0)	2016 (48.8)	1040 (50.5)	9 (60.0)
35–44	514 (11.2)	23 (8.5)	1 (50.0)	468 (11.3)	217 (10.5)	0 (0.0)
45–54	7 (0.2)	0 (0.0)	0 (0.0)	7 (0.2)	2 (0.1)	0 (0.0)
55–64	0 (0.0)	0 (0.0)	0 (0.0)	0 (0.0)	0 (0.0)	0 (0.0)
≥65	0 (0.0)	0 (0.0)	0 (0.0)	0 (0.0)	0 (0.0)	0 (0.0)
Insurance^a^						
Medicaid	3075 (67.2)	191 (70.2)	2 (100.0)	2906 (70.4)	1452 (70.5)	13 (86.7)
Medicare	75 (1.6)	6 (2.2)	0 (0.0)	89 (2.2)	37 (1.8)	0 (0.0)
Private	742 (16.2)	36 (13.2)	0 (0.0)	705 (17.1)	338 (16.4)	0 (0.0)
Other	687 (15.0)	39 (14.3)	0 (0.0)	429 (10.4)	234 (11.4)	2 (13.3)
Clinical characteristics						
Tested for gonorrhea, chlamydia, or trichomoniasis						
Yes	986 (21.5)	204 (75.0)	1 (50.0)	1040 (25.2)	737 (35.8)	5 (33.3)
No	3593 (87.5)	68 (25.0)	1 (50.0)	3089 (74.8)	1324 (64.2)	10 (66.7)
Presenting symptoms by *ICD-10* code						
STI related	224 (5.2)	57 (22.1)	1 (50.0)	261 (6.9)	161 (8.6)	0 (0.0)
Opioid use	3 (0.1)	0 (0.0)	0 (0.0)	8 (0.2)	4 (0.2)	0 (0.0)
Any substance use	44 (1.0)	5 (1.8)	1 (50.0)	63 (1.5)	36 (1.7)	0 (0.0)
Abdominal and pelvic pain	920 (20.1)	71 (26.1)	0 (0.0)	1053 (25.5)	566 (27.5)	7 (46.7)
Rashes	10 (0.2)	0 (0.0)	0 (0.0)	16 (0.4)	6 (0.3)	0 (0.0)
Flu-like illness	61 (1.3)	5 (1.8)	0 (0.0)	28 (0.7)	10 (0.5)	0 (0.0)
Altered mental status	16 (0.3)	2 (0.7)	0 (0.0)	37 (0.9)	10 (0.5)	0 (0.0)
Additional blood draw^b^						
Yes	3356 (73.3)	223 (82.0)	1 (50.0)	3329 (80.6)	1846 (89.6)	11 (73.3)
No	1223 (26.7)	49 (18.0)	1 (50.0)	800 (19.4)	215 (10.4)	4 (26.7)
Acuity level by Emergency Severity Index						
1	7 (0.2)	3 (1.1)	0 (0.0)	15 (0.4)	7 (0.3)	0 (0.0)
2	798 (17.4)	31 (11.4)	0 (0.0)	645 (15.6)	296 (14.4)	2 (13.3)
3	2987 (65.2)	185 (68.0)	2 (100.0)	2829 (68.5)	1479 (71.8)	11 (73.3)
4	549 (12.0)	39 (14.3)	0 (0.0)	292 (7.1)	101 (4.9)	0 (0.0)
5	28 (0.6)	1 (0.4)	0 (0.0)	24 (0.6)	6 (0.3)	0 (0.0)
Unknown	210 (4.6)	13 (4.8)	0 (0.0)	324 (7.8)	172 (8.3)	2 (13.3)
HIV testing						
Not tested for HIV	3901 (85.2)	8 (2.9)	0 (0.0)	2047 (49.6)	33 (1.6)	0 (0.0)
HIV negative	677 (14.8)	264 (97.1)	2 (100.0)	2075 (50.3)	2021 (98.1)	14 (93.3)
Known HIV infection	0 (0.0)	0 (0.0)	0 (0.0)	5 (0.1)	5 (0.2)	0 (0.0)
New diagnosis of HIV	1 (0.0)	0 (0.0)	0 (0.0)	2 (0.0)	2 (0.1)	1 (6.7)

Abbreviations: ED, emergency department; HIV, human immunodeficiency virus; *ICD-10*, *International Classification of Diseases, Tenth Revision*; PAI, presumed active syphilis infection; STI, sexually transmitted infection.

^a^For insurance, other includes self-pay and unknown insurance information.

^b^Additional blood draw refers to any laboratory test requiring a blood draw other than HIV or syphilis.

For women under age 50, screening rates (4.5% encounters pre, 29.6% post) and syphilis cases (27 pre, 142 post) increased post-intervention, in parallel with trends among the larger ED population. Post-intervention demographic and clinical trends among this group largely mirrored the entire study population. Notably, around half this population did not receive a pregnancy test in the ED, including 35.3% of the screened population post-intervention, and 38.0% of PAI post-intervention. Among female PAI under age 50, a large proportion (76.8%) used Medicaid or were uninsured (12.0%), and 24.6% of PAI had no additional laboratory test requiring a blood draw other than an HIV or syphilis test. A minority of this subgroup of PAI were tested for urogenital STIs (25.4%) or presented with STI-related complaints (13.5%).

## DISCUSSION

This study demonstrates that a routine, opt-out syphilis screening program in the ED can have a large effect on both screening and diagnosis rates, both in the ED population as a whole, and importantly among key demographics such as pregnant persons, who are a priority group for enhanced syphilis screening and treatment. Introduction of an opt-out screening program increased the numbers of non-Hispanic Black and Hispanic individuals screened and resulted in a racial and ethnic distribution of the screened population that was more in line with that of the general ED population. While the rise in syphilis rates post-intervention may be attributable in part to secular trends, more frequent and more equitable screening will be important to address this important epidemic. The number needed to screen to detect one infection needing treatment was only 75, considerably lower than estimates for HIV screening in a similar population [16], suggesting that this intervention may be efficient in similar populations.

Although the proportion of females screened remained almost identical between both periods, sex differences among PAI decreased, with females representing 42.5% of PAI post-intervention, higher than the 24.8% of primary and secondary syphilis represented by women nationally [1]. This may simply reflect a rapid recent increase in syphilis among women [17], although interpretation of this finding in the setting of secular trends is limited by lack of stage information in the present study. These results may also suggest that opt-out screening could help mitigate some implicit bias affecting which populations are ultimately screened in the ED, allowing for more gender equity in screening. Importantly, regardless of other demographics, after implementation of universal screening, only a small proportion of PAI presented with STI symptoms or underwent urogenital STI testing. Not only has this model shown promise at reaching key demographic groups and asymptomatic patients, but it has also proven to be sustainable over time, continuing to screen and diagnose ED patients at similar rates for more than 5 years.

The optimal model for syphilis screening has not yet been identified, particularly with respect to prevention of congenital syphilis. Previous literature has examined targeted syphilis screening for patients undergoing urogenital STI testing [13], pregnant patients [9], patients with identified “risk factors,” [10, 12] or some combination of the above [11]. While all of these strategies resulted in modest increases in both screening and diagnosis among their intended target populations, the present study is the first to examine an opt-out strategy. In this study, a universal testing prompt increased screening and diagnosis dramatically among pregnant women, more so than in the rest of the population, suggesting this as a highly effective strategy that has the potential to impact the congenital syphilis epidemic in the US. An additional 127 diagnoses came from nonpregnant women of reproductive age, demonstrating that a limited screening approach targeted to pregnant women could potentially miss key opportunities to screen and treat women before pregnancy, as well as identify cases among their partners or potential partners. Additionally, an approach targeted to pregnant women is unlikely to reach men who have sex with men (MSM), a priority group with a high prevalence of syphilis in the US [1].

The question of targeted versus universal screening has been addressed extensively in the ED HIV screening literature. Multiple studies have shown no major differences in positivity or screening rate between the 2 models [18, 19], and universal HIV screening has been shown to be cost-effective [7]. The evidence suggests that attempts at risk stratification for HIV screening in the ED may actually lead to lower uptake of screening [20], which may be due to perceived workflow constraints or stigma associated with risk factor questionnaires [21], and that patients declining routine HIV screening may have higher rates of HIV [22, 23]. For all these reasons, the CDC recommends universal HIV screening in high-prevalence areas [5]. Syphilis is similar to HIV in many key ways: It disproportionately affects very similar populations, it can remain asymptomatic for long periods of time, and it can lead to serious and sometimes fatal complications. The present study's finding that few PAI in the post-intervention period presented with STI symptoms or were tested for urogenital STIs suggests that the universal rather than symptom-based approach is necessary to avoid missed syphilis diagnoses. Additionally, although rash is a common presenting symptom of syphilis, as noted in the high rate of rash presentations among PAI pre-intervention, few individuals screened post-intervention presented with rash, suggesting that a universal approach could improve diagnosis by mitigating some of the lack of awareness of the common presentations of syphilis among clinicians. As the weight of the evidence supports universal HIV screening in EDs in high-prevalence communities, a similar strategy should be strongly considered for syphilis [8]. Additionally, although many routine screening programs are built to add screening to existing blood draws for simplicity of implementation, this study found that 17.3% of PAI had no additional blood draw besides HIV or syphilis, a finding that suggests screening programs should consider including patients regardless of other blood draws, at least in certain scenarios, such as pregnancy or presentation for STI symptoms. While the association between the presence of an additional blood draw and the likelihood of syphilis screening suggests that tying screening to other blood draws will facilitate screening, this strategy also risks missing a large proportion of diagnoses.

As more sites consider implementation of similar screening programs, it will be important to define the optimal parameters for screening, which may vary by location. Although this program was designed not to include individuals over age 65 in routine screening in order to leverage existing HIV screening infrastructure, it did result in a modest increase in screening in this group, with 7.7% of diagnoses among those over age 65. Almost a third of these had altered mental status, suggesting that a targeted approach may be useful, but because screening in this population was not universal, further research is needed to assess the utility of universal screening in the elderly. Additionally, the screening model described here excluded PWH when identified by the EHR, as it was built upon historical HIV screening infrastructure. Despite this, 9.9% of PAI were PWH, suggesting that this population should be included in ED syphilis screening programs in the future, even though many are screened routinely for syphilis as part of their HIV care. Concurrent diagnoses of HIV and syphilis also increased significantly post-intervention, further supporting the need for a combined screening HIV and syphilis model.

### Limitations

This was a single site study, and additional research is needed to determine if these findings are generalizable to other EDs. As this analysis included EHR data abstraction, certain aspects were unable to be determined, including the reason for syphilis testing during the post-intervention period, and in some cases, clinical history. This study was not able to identify the stage of syphilis infection, which may limit the ability to interpret the results, especially in the setting of secular trends of rapidly increasing syphilis, particularly among women. This screening model did not include PWH and individuals over age 65, so further study will be needed in these populations to fully establish the optimal inclusion criteria for future screening models.

## CONCLUSIONS

With the recent dramatic rise in syphilis and congenital syphilis, there is an urgent need to identify strategies to increase screening and early diagnosis among priority populations, especially those with limited access to care, including limited or no prenatal care. This study demonstrates that the introduction of opt-out syphilis screening in the ED can have profound effects on screening and diagnosis rates among racial and ethnic minorities, those holding public insurance, women, and most strikingly, pregnant women. With most syphilis cases identified among individuals without STI symptoms, an opt-out model may represent an ideal screening approach that is effective not just in reaching women who are already pregnant, but to screen women and their partners before pregnancy even occurs, as well as to reach other populations with high syphilis prevalence, such as MSM and PWH. While more research is needed to evaluate the optimal model and frequency of ED syphilis screening, a universal approach to screening represents a highly effective and feasible strategy to address the syphilis epidemic in high-prevalence areas in the US.

## Notes

***Author contributions.*** K. A. S., A. H., and J. S. conceived the study and designed the trial. K. A. S. and E. A. extracted the data. E. F., J. M., and K. A. S. designed the statistical analysis plan and analyzed the data. K. A. S. drafted the manuscript, and all authors contributed substantially to its revision. K. A. S. takes responsibility for the manuscript as a whole.

***Acknowledgments.*** This work was supported by the National Institute of Allergy and Infectious Diseases of the National Institutes of Health (award number K23AI166277).

***Data availability.*** Data are not publicly available.
